# Prevalence of consumption of mechanically separated meat, consumer profile, nutrient intake and food choices among manufacturing workers in Northeastern Brazil

**DOI:** 10.1038/s41598-024-64247-6

**Published:** 2024-06-10

**Authors:** Raiane Medeiros Costa, Antonio Gouveia Oliveira, Anissa Melo de Souza, Karina Gomes Torres, Gabriela Santana Pereira, Ingrid Wilza Leal Bezerra

**Affiliations:** 1 Postgraduate Program in Health Sciences, Centro de Ciências da Saúde, Universidade Federal do Rio Grande do Norte, Natal, RN Brazil; 2 Pharmacy Department, Centro de Ciências da Saúde, Universidade Federal do Rio Grande do Norte, Natal, RN Brazil; 3 Nutrition Department, Centro de Ciências da Saúde, Universidade Federal do Rio Grande do Norte, Natal, RN Brazil

**Keywords:** Mechanically separated meat, Meat products, Ultra-processed food, Meat industry, Nutrient consumption, Food choices, Health care, Nutrition, Public health

## Abstract

Mechanically separated meat (MSM) is widely used in the food industry, however, there is a lack of studies on its consumption in populations. The objective of this study was to identify the frequency and amount of MSM consumption, factors associated with MSM consumption, nutrient intake and preferential choice of food groups among MSM consumers. This was an observational, cross-sectional prospective study based on a probability sample of manufacturing workers, conducted in Brazil. Logistic and linear multiple regression with robust standard errors were used. 921 workers from 33 manufacturing companies were studied, with an average age of 38.2 ± 10.7 years, 55.9% males. MSM products are consumed by 28.8% and represent in average 10% of total daily caloric intake, and 47.3% of the daily kcal from ultra-processed products. Younger age and greater waist circumference are associated with MSM consumption. Younger age and lesser educational level are associated with increased contribution of MSM to total daily kcal intake. MSM consumers have greater consumption of energy, fats, carbohydrates and sodium. Their dietary patterns are characterized by lower consumption of in natura and minimally processed foods, such as tubers and roots, fruits, white and red meat, and eggs and greater consumption of ultra-processed foods and beverages.

## Introduction

Mechanically separated meat (MSM) is a raw, paste-like material, obtained by mechanical separation of soft tissue residues from bones, which remain after cutting and puncture of carcasses of poultry, mutton, pork and bovine origin. MSM is used as an ingredient in a variety of meat products and may have a significant impact on their quality, as well as on consumer satisfaction and safety^1^. Due to its low cost, MSM is widely used in the food industry^2^ and is commonly used in meat processing around the world, mainly in cooked or roasted meat products, canned meat, homogenized sausages, frankfurters, bologna, nuggets, patties and many other ready-to-eat ultra-processed food products^3,4^.

Identifying and differentiating MSM from fresh meats, minced meats and meat preparations is important because they are food products with different characteristics, properties and processing levels. For the European Commission, MSM is “the product obtained by removing meat from fleshy bones after deboning or from poultry carcasses, using means that result in the loss or modification of the structure of the muscle fiber”^5^. In the United States, mechanically separated meat is a paste-like and batter-like meat product produced by forcing bones with attached edible meat under high pressure through a sieve or similar device to separate the bone from the edible meat tissue. Due to FSIS (Food Safety and Inspection Service) regulations enacted in 2004 to protect consumers against Bovine Spongiform Encephalopathy, mechanically separated beef was considered inedible and was prohibited for use as human food^6^. In Brazil, MSM is defined as meat obtained by a mechanical grinding and separation process of bones from butchered animals (poultry, bovine and pork), intended for the production of specific meat products. Furthermore, only bones, carcasses or parts of carcasses of butchered animals that have been approved for human consumption by the Federal Inspection Service can be used^7^. Some aspects of conservation under freezing, physical–chemical criteria (percentage of protein, fat, calcium and bone diameter) and microbiological criteria were added in the change to this normative instruction in 2020^8^. According to this regulation, in Brazil, MSM must have at least 12% protein and a maximum of 30% fat and 1.5% calcium content (dry basis).

In the production of MSM, after the primal meat cuts are extracted, the remaining traces of meat are removed to obtain as much meat as possible. When mechanical methods are used for separating the meat from bones, the carcasses are forced, under high pressure, through a sieve that separates bone fragments from the meat tissue and some bone particles inevitably pass into the final product^9^. The quality, as well as the usefulness of processing this raw material largely depends on the technique through which it is obtained^10^. Two mechanical methods of boning processing are recognized, high pressure or low pressure. High pressure MSM, the most frequently used type, is characterized by a greater degree of destruction of muscle fibers, which makes it more perishable, of poorer technological quality and less stability compared to manually boned meat, i.e. in the traditional way^11,12^.

Being a cheaper product than hand-boned meat, the noticeable increase over time in the manufacturing and marketing of this type of food product is readily understandable. Consumer tastes have been changing, from a preference for whole animal carcasses to cut parts and processed products, mainly chicken meat (which has the largest share in the meat production industry). This has led to an increase in the availability of bones and poultry trimmings that remain after the larger muscles (breasts and thighs) are separated. These parts still contain portions of muscle tissue, most of which can be recovered through the production of MSM^10,13–15^. Thus, MSM emerged in the food industry as an alternative to the disposal of those materials and an opportunity to use them for the production of by-products, resulting in semi-finished or ready-made products with good commercial properties, long shelf life and an acceptable price^16^.

However, the composition of these reconstituted meat products using mechanical deboning include a high fat content, propensity to lipid oxidation and a series of spices and additives used in the food industry with the intention of stabilizing and increasing the shelf life of these products that have a non-standard composition and who are prone to microbiological risks^13,17,18^. It is known that during meat processing, more meat and animal fat are added along with a wide range of non-meat substances and additives, leading to more complex products. Although the differences between types of meat (red, white, different cuts, processed meats, etc.) may not be clear to many consumers, the differential benefits and risks of developing chronic diseases due to its consumption are significant^18^. In the processing, there is extreme mechanical stress, extraction of a high proportion of fat and heme components (hemoglobin and myoglobin) from the bone marrow, resulting in a pasty consistency and conditions that may lead to lipid oxidation problems and color instability of the final product^13^. In addition, the bones used for the production of MSM may contain a high number of microorganisms^3,19,20^. Thus, several methods are used to stabilize these food products, among them is sodium nitrite which is used in industrial practice for the curing process, which plays a role in color formation and also contributes to the flavor of products containing MSM^21^.

This food transformation process aims to stabilize food products as safe and suitable for consumption, extend the shelf life, enhance less attractive and/or less sought after-cuts, diversify the market offer, and propose products that are easy to cook and store, tasty, or even ready-to-eat foods^22^. Thus, the production of MSM made it possible to use considerable quantities of parts that have lower commercial value (bones, carcasses…), transforming them into products with greater added value and high sales potential, attractive prices in relation to other types of meat, and lower processing costs^23^. All these characteristics have been driving its growth and increased sales throughout the world, represented by numerous types of products and brands, such as the sausages and frankfurters that are the preference of MSM consumers as seen in this study, used in preparations as hot dogs, hamburgers, feijoada, industrialized pâtés, mortadella, nuggets, regional corn-based foods, frozen ready meals, among others, which are widely sold in retail chains in Brazil.

In recent years, research related to the consumption of ultra-processed foods in general has been explored in the literature, but little has been studied specifically about MSM. Existing studies have focused on microbiological aspects, methods of identification, technological applications, and rarely if ever have highlighted their nutritional aspects and characteristics related to their food consumption by the population. Therefore, the aim of the present study was to quantify the frequency and amount of MSM consumption, to identify indicators and determinants of MSM consumption, the association between MSM consumption and nutrient intake, and the relationship with food groups and food classes according to the level of processing. The focus of our research is on manufacturing workers, who are an important segment of the population, in most countries representing more than 10% of the working force and accounting for more than 10% of the Gross National Product^24^, in whom nutrition directly influences their health and productivity, but who may be in a position of vulnerability to potential lack of access to healthy food in the workplace, as studies have shown^25^.

## Materials and methods

### Study design and population

This was an observational, cross-sectional study based on a representative, probability sample of manufacturing workers in the state of Rio Grande do Norte, Brazil. The sampling plan consisted of a combined stratified proportional and two-stage survey. The strata were company size, with three levels (small, less than 50 workers; medium, 50 to 500 workers; and large, more than 500 workers), and sector of activity, also with three levels (food and beverages, non-metallic minerals, and textiles) corresponding to the industries with greater representation in the state.

For the first sampling stage, manufacturing companies were selected in each combination of strata levels, in a number proportional to the total number of companies in the state in that combination of strata, by simple random sampling from a sampling grid provided by the federation of state industries (FIERN). In the second sampling stage, a fixed number of workers from each company recruited in the first stage was selected by simple random sampling, using a computer random number generator, from listings of workers provided by the human resources departments of each company.

All companies in the state from the three mentioned sectors of activity that agreed in written to participate in the research were eligible for the study. The inclusion criteria for workers were: over 18 years old with an effective employment relationship with the company. Pregnant women, temporary employees, interns and employees on probationary periods were excluded from the research.

The research was approved by the Research Ethics Committee of the Hospital Universitário Onofre Lopes (authorization number 2.198.545/2017) and carried out in accordance with the Declaration of Helsinki. All participants gave informed consent in writing.

### Investigation

In the preparation of the field survey, each visit of the research team was scheduled with each company to occur between Tuesdays and Saturdays. The workers that were previously selected were approached during lunch time and invited to participate in the research. After signing the informed consent form, biodemographic data (age, sex, marital status, education, income, participation in in-house training programs) were recorded and food consumption was assessed using the 24 h dietary recall method (24HR). For the application of the 24HR, the interviewers used the Multiple Pass Methods—MPM, which helps the interviewee to remember the foods consumed the day before the interview and, therefore, minimizes errors in food consumption measurements. This Method is divided into stages, which include a list of the foods consumed, time and place of the meal, detailed description of the quantities of the food (in household measurements), preparation and cooking methods, among other aspects^26^.

Body mass index (BMI), defined by weight in kg divided by squared height in meters, was measured with a digital scale (Inner Scan, Tanita Corp., Tokyo, Japan) and a body height meter (Sanny, São Bernardo do Campo, SP, Brazil). Waist circumference (WC), measured at the midpoint between the lower edge of the last rib and the iliac crest, was measured with a tape meter (Cescorf, Ltda, Porto Alegre, RS, Brazil). The measurements were performed according to the guidelines of the Brazilian Alimentary and Nutritional Surveillance System (SISVAN)^27^.

### Characterization of food consumption

The descriptions of food consumption extracted from the 24HR obtained from home measurements were quantified in units of weight and volume, using previously established references (direct weighing of food, photographic records and specific manuals for this purpose^28^. The analysis of the nutritional composition of workers’ food consumption was carried out using reference Food Composition Tables^29–31^ supplemented, when necessary, with information from food labels.

The foods registered in 24HR were classified in two ways: according to the NOVA^32^ group and according to the food group, following the classification of food groups in the Food Guide for the Brazilian Population, which considers biological, physicochemical, organoleptic and dietary characteristics, aggregating foods into sets that have similar culinary uses and nutritional profiles^33,34^. Then, the sets defined by the Food Guide were disaggregated into 44 food groups, based on the diversity of foods and preparations referred to in the 24HR. Most freshly prepared foods, which include items from different food groups, were described in their ingredients, defining quantities based on a Technical Preparation Sheet. A small number of freshly prepared mixed foods, based mainly on fresh and/or minimally processed foods, typical of Brazilian cuisine, were classified in the group of foods with the highest contribution of ingredients. Detailed information about the criteria used for this classification was published in another study^35^.

### Statistical analysis

The svy suite of commands of the Stata^®^ 15.1 statistical software (Stata Corporation, College Station, TX, USA) was used for data analysis. The statistical analysis considered the stratification by company size and activity sector, the two-stage sampling plan with companies as the primary sampling units and the workers nested within companies. All analyses were weighted, with sampling weights computed according to the survey plan, a finite population correction was included in the calculations, and robust standard errors were used. Unless otherwise stated, the displayed results are population estimates, not sample statistics, and point estimates with 95% confidence intervals (95%CI) are presented throughout. Associations of consumption of MSM with binary and continuous worker variables were analyzed with logistic regression and multiple linear regression, respectively. Differences in nutrient and food groups consumption between MSM consumers and non-consumers were analyzed with multiple linear regression. A p-value < 0.05 was considered evidence of statistical significance.

## Results

From September 2017 to July 2018, 921 workers from 33 manufacturing companies were enrolled in the study. The sectors of activity of the surveyed companies were food and beverages (14 companies), minerals (6 companies) and textiles (13 companies). Thirteen companied were small-sized, 14 midium-sized, and 6 large-sized. There were no refusals to participation in the study and there were no missing or incomplete data. The average interview time with each worker was under 15 min. Study data are available from the online version (Supplementary Information).

Table 1 presents the descriptive statistics of the study sample, as well as the estimates derived for the target population using sampling weights and the characteristics of the survey design (two-stage clustered sampling and stratification). The population of manufacturing workers had a mean age of 38.6 years, 53.6% were males, 62.6% lived with a companion, the average income was 1.53 minimum wages, the average body mass index was 27.4, 33.2% had normal weight, 38.2% were overweight and 27.5% were obese.

**Table 1:** Characteristics of the study sample and population estimates.

Variable	Sample statistics	Population estimates
Age, years	38.2 ± 10.7	38.6
Sex, male	515 (55.9%)	53.6%
Social support	577 (62.7%)	62.6%
Children	638 (69.3%)	70.6%
Education > high school	527 (57.3%)	60.3%
Income, minimum wages	1.46 ± 1.61	1.53
In-house formation	172 (18.7%)	20.1%
Body Mass Index	27.2 ± 4.80	27.4
Waist Circumference, cm	89.7 ± 11.9	90.4
Diagnosis		
Underweight	12 (1.30%)	1.10%
Normal weight	316 (34.4%)	33.2%
Overweight	359 (39.0%)	38.2%
Obesity I	178 (19.4%)	20.4%
Obesity II	40 (4.35%)	5.11%
Obesity III	15 (1.63%)	1.94%

Tabulated values are mean ± standard deviation, or number(percent).

Table 2 presents indicators of the daily consumption of MSM. About 29% of the population (95%CI 24.5%-33.5%) include MSM in the diet, at an average daily intake of 65.7 g, corresponding to a daily total of 202.5 kcal. Kilocalories from MSM represent 9.95% (95%CI 8.68%-11.2%) of total daily caloric intake, and nearly half (47.3%) of daily kcal from ultra-processed (UP) products comes from MSM.

**Table 2:** Indicators of consumption of Mechanically separated meats (MSM).

Consumption of MSM	Mean/percent	95% confidence interval
Consumers of MSM	28.8%	24.5%–33.5%
Daily consumption of MSM (g)	65.7	58.4–73.1
Daily consumption of MSM (Kcal)	202.5	176.7–228.3
Contribution of MSM to total daily Kcal	9.95%	8.68%–11.2%
Contribution of MSM to total daily Kcal from UP	47.3%	42.3%–52.4%

Table 3 presents, for each socioeconomic characteristic of the subjects, the prevalence of MSM consumption, the consumption of MSM as percent contribution to total daily calories, and as percentage of the total calories from UP products. Persons with lower literacy incorporate a greater amount of MSM in their diet (p = 0.05) and in their share of UP products (p = 0.04). Male workers may have greater tendency to consume MSM products, but the difference did not quite reach statistical significance (p = 0.06). Increased consumption of MSM is also associated with younger age (odds-ratio 0.970, p = 0.004) but neither with BMI (odds-ratio 1.009, p = 0.62) nor waist circumference (WC) (odds-ratio 1.007, p = 0.37). Age correlates with percent contribution of total caloric intake (regression coefficient − 0.070, p = 0.007), but no correlation was found with BMI (regression coefficient 0.054 p = 0.28) and WC (regression coefficient 0.013, p = 0.49). Neither variable was correlated with percent contribution of total UP consumption (regression coefficients: age − 0.213, p = 0.07; BMI 0.273, p = 0.28; WC 0.090, p = 0.31). In multivariate analysis, age (OR 0.964, p = 0.002) and waist circumference (OR 1.017, p = 0.05) were associated with MSM consumption. The variables independently associated with MSM percent contribution to total calorie intake were age (regression coefficient − 0.089, p = 0.002) and lower education (regression coefficient 1.569, p = 0.01). In multivariate analysis the variables associated with MSM percent contribution to total UP Kcal consumption, were age (regression coefficient − 0.294, p = 0.020) and lower education (regression coefficient 6.560, p = 0.012).

**Table 3:** Determinants of consumption of mechanically separated meats (MSM).

	Prevalence of MSM		MSM % of total Kcal consumption		MSM % of total UP Kcal consumption	
	%	p-value	Mean	p-value	Mean	p-value
Sex						
Female	24.7	**0.06**	11.8	0.84	52.5	0.62
Male	32.3		8.71		43.9	
Social support						
Single	25.6	0.18	10.3	0.44	50.4	0.53
Married	30.7		9.79		45.8	
Children						
No	29.8	0.75	10.5	0.53	43.8	0.66
Yes	28.4		9.71		48.9	
Education						
< = 2	30.5	0.46	11.5	**0.05**	53.4	**0.04**
> 2	27.8		8.80		42.9	
Income						
< = 1 min. wage	29.3	0.75	10.9	0.14	52.3	0.08
> 1 min. wage	28.2		8.74		41.3	
Formation						
No	28.3	0.58	10.2	0.88	49.0	0.58
Yes	30.9		9.03		41.2	

Significants values are in bold.

In Table 4, the daily intake of nutrients was compared between consumers and non-consumers of MSM. There was a statistically significant association between consumption of MSM and increased intake of energy (p = 0.002), carbohydrates (p = 0.01), total lipid (p < 0.001), saturated (p = 0.006) and monounsaturated fat (p < 0.001), omega-6 fatty acids (p = 0.007), sodium (p < 0.001) and fiber (p = 0.029).

**Table 4:** Daily intake of nutrients in non-consumers and consumers of mechanically separated meats (MSM).

Nutrient	No MSM	MSM	difference	95% CI		p-value
	Mean	Mean				
Kilocalories	1922.6	2198.2	275.6	118.0	433.2	**0.002**
Carbohydrates (Kcal)	1017.8	1137.1	119.2	33.0	205.5	**0.010**
Protein (Kcal)	383.7	395.4	11.8	− 20.3	43.9	0.45
Lipid (Kcal)	520.9	662.0	141.2	80.3	202.0	** < 0.001**
Saturated fat (Kcal)	175.5	207.8	32.2	10.6	53.9	**0.006**
Monounsaturated fat (g)	17.1	21.9	4.79	2.8	6.83	** < 0.001**
Polyunsaturated fat (g)	13.3	14.9	1.61	− 1.1	4.36	0.23
Omega-6 fatty acids (g)	10.7	12.7	2.01	0.6	3.37	**0.007**
Omega-3 fatty acids (g)	1.18	1.47	0.28	0.0	0.58	0.058
Trans fatty acids (g)	2.12	2.33	0.21	− 0.2	0.62	0.28
Fiber (g)	24.4	27.2	2.82	0.3	5.31	**0.029**
Calcium (mg)	458.1	481.8	23.7	− 53.3	100.8	0.52
Copper (mg)	1.31	1.24	− 0.07	− 0.5	0.39	0.75
Iron (mg)	10.3	10.7	0.32	− 1.1	1.73	0.64
Magnesium (mg)	237.9	251.0	13.0	− 10.2	36.2	0.25
Manganese (mg)	2.52	2.42	− 0.11	− 0.7	0.46	0.69
Phosphorus (mg)	1159.5	1238.8	79.4	− 26.9	185.7	0.13
Potassium (mg)	2269.1	2440.0	170.9	− 4.2	346.0	0.055
Selenium (mg)	82.9	77.5	− 5.37	− 15.6	4.88	0.28
Sodium (mg)	4059.0	5147.3	1088.3	706.3	1470.3	** < 0.001**
Zinc (mg)	11.3	12.4	1.07	− 0.3	2.46	0.12
Vitamin A (mg)	704.2	569.4	− 134.9	− 576.5	306.7	0.53
Vitamin C (mg)	137.3	135.0	− 2.25	− 54.1	49.6	0.93
Vitamin D (mg)	2.56	2.55	− 0.01	− 0.7	0.68	0.97
Vitamin E (mg)	4.94	4.39	− 0.55	− 1.5	0.40	0.23
Cobalamin (mg)	6.36	5.60	− 0.76	− 3.3	1.83	0.54
Niacin (mg)	20.0	19.8	− 0.20	− 2.6	2.14	0.86
Pyridoxine (mg)	0.93	0.96	0.04	− 0.1	0.22	0.68
Riboflavin (mg)	1.13	1.25	0.12	**− **0.1	0.32	0.24
Thiamine (mg)	1.10	1.21	0.11	0.0	0.24	0.089

Significants values are in bold; CI: confidence interval.

Table 5 shows the food products containing MSM that are most consumed, both as total daily energy and as percent of total MSM consumption. Sausages and frankfurters are the preferred products, each representing over one-third of MSM products consumption.

**Table 5:** Sources of Mechanically separated meats (MSM).

Food	Daily consumption (Kcal)		Percent contribution to total MSM consumption	
	Mean	95% CI	Mean	95% CI
Hamburger	14.5	5.41–23.6	6.36	2.20–10.5
Sausage	96.9	72.1–121.7	38.3	30.9–45.8
Mortadella	25.9	13.1–38.7	19.6	13.2–26.0
Frankfurter	61.6	46.5–76.7	33.8	27.1–40.4
Other	3.54	0.01–7.19	1.90	0.01–4.24

﻿CI: confidence interval.

Table 6 presents the comparison, between MSM consumers and non-consumers, of the consumption of food products from NOVA classes of level of processing. MSM consumers showed a lesser intake of unprocessed or minimally processed foods (p < 0.001), and a higher intake of ultra-processed foods and drink products (p < 0.001).

**Table 6:** Consumption of NOVA class in percentage of total energy intake.

NOVA group foods	No MSM		MSM		Difference	95% CI	p-value
	Mean	S.E	Mean	S.E			
Unprocessed or minimally processed foods	67.7	0.94	58.0	1.55	− 9.74	− 13.6; − 5.93	** < 0.001**
Processed foods	16.8	0.73	16.4	1.19	− 0.34	− 3.26; 2.57	0.81
Ultra-processed foods and drink products	15.5	0.70	25.6	1.35	10.1	6.79 ;13.4	** < 0.001**

Significants values are in bold; MSM: mechanically separated meat; S.E.: standard error; ﻿CI: confidence interval.

Table 7 presents the difference between consumers and non-consumers of MSM in the composition of the diet, expressed as percent of total energy intake of food groups defined according to their nature and nutritional profile. Food groups representing less than 1% of total energy intake are not shown. MSM consumers have increased intake of corn (p = 0.001) and processed meats (p < 0.001), and lesser intake of tubers and roots (p = 0.017), fruits (p = 0.015), white (p > 0.001) and red meat (p = 0.004), and eggs (p = 0.006).

**Table 7:** Consumption of food groups in percentage of total energy intake.

Food group	No MSM		MSM		Difference	95%	CI	p-value
	mean	S.E	mean	S.E				
Beans	5.51	0.22	5.78	0.36	0.27	− 0.64	1.18	0.54
Rice	6.95	0.32	6.12	0.40	− 0.83	− 1.85	0.19	0.10
Corn	4.94	0.35	7.95	0.61	3.01	1.51	4.51	**0.001**
Whole grains	1.48	0.25	1.38	0.49	− 0.10	− 1.29	1.09	0.86
Breads	8.38	0.50	7.51	0.66	− 0.87	− 2.60	0.86	0.30
Pasta	4.53	0.36	5.01	0.63	0.49	− 1.02	1.99	0.50
Biscuits	4.86	0.45	5.37	0.78	0.51	− 1.40	2.42	0.58
Tubers and roots	7.47	0.56	5.25	0.58	− 2.22	− 3.98	-0.46	**0.017**
Vegetables	1.07	0.06	1.01	0.09	− 0.06	− 0.30	0.18	0.61
Soups	1.15	0.18	0.80	0.23	− 0.36	− 0.95	0.23	0.22
Fruits	3.23	0.26	2.24	0.26	− 0.99	− 1.76	− 0.22	**0.015**
Fruit juices	0.96	0.08	1.02	0.09	0.06	− 0.20	0.31	0.65
Milk and Dairy	6.51	0.37	5.91	0.67	− 0.60	− 2.19	1.00	0.44
Red meat	9.11	0.49	6.46	0.57	− 2.65	− 4.31	− 0.99	**0.004**
White meat	10.37	0.52	6.74	0.50	− 3.63	− 5.18	− 2.08	** < 0.001**
Eggs	3.09	0.27	1.81	0.29	− 1.28	− 2.15	− 0.42	**0.006**
Processed meats	0.15	0.04	9.93	0.64	9.78	8.41	11.15	** < 0.001**
Sugary drinks	0.66	0.13	1.01	0.19	0.35	− 0.10	0.80	0.12
Coffee	1.14	0.04	1.23	0.14	0.09	− 0.24	0.42	0.57
Fast food	0.88	0.32	2.23	0.59	1.35	− 0.10	2.80	0.066
Fried and baked snacks	2.06	0.33	1.81	0.42	− 0.25	− 1.40	0.90	0.65
Sweets and desserts	1.89	0.23	2.41	0.42	0.52	− 0.47	1.51	0.28
Sugar	4.40	0.22	4.44	0.29	0.04	− 0.76	0.85	0.91
Margarine	1.16	0.13	1.01	0.14	− 0.16	− 0.57	0.26	0.43
Oils	3.15	0.14	2.87	0.19	− 0.28	− 0.77	0.22	0.25
Cakes	1.90	0.29	1.33	0.29	− 0.57	− 1.46	0.32	0.19

Significants values are in bold﻿; MSM: mechanically separated meat;. S.E.: standard error; ﻿CI: confidence interval.

## Discussion and conclusions

To the best of our knowledge, this is the first study analyzing the consumption of MSM in the population. The results of this study on Brazilian manufacturing workers have shown that MSM consumption represents around 10% of individuals’ total daily caloric intake, and almost half of the daily kcal from ultra-processed products (UP). Its consumption is greater among younger people, people with lower education, and possibly also in males. Individuals who consume MSM have greater consumption of energy, carbohydrates, fats (total, saturated, monounsaturated and omega-6 fatty acids), sodium and fiber. Their dietary patterns are characterized by lower consumption of in natura foods or minimally processed foods, such as tubers and roots, fruits, white and red meat and eggs, and greater consumption of ultra-processed foods and beverages. The MSM most consumed were sausages and frankfurters.

This type of meat product is only present in ultra-processed foods (within the group of processed meats), and in this study we observed that MSM correspond to almost half of UP consumption. Therefore, knowing that the consumption of UP, which already has a big expression in high-income developed countries and has been increasing rapidly in middle-income countries such as Brazil^36^, an increasing trend in the consumption of MSM can be implied.

The explanations for the differences in MSM consumption between sexes, age groups and education may be because, being a UP food, MSM is likely to be mostly consumed by individuals who also have UP foods in their dietary habits, and studies have shown that UP consumption is related to younger age and male sex^37^. Socioeconomic inequality, including a lower educational level, may lead to a preference for less expensive, less nutritious foods^38^. No studies have yet investigated variables associated with the consumption of MSM, but the literature on the broader class of UP foods confirms our findings. In a study in Canada, with around 19 thousand adults and with a similar methodology to our study (24HR as a dietary survey), the consumption of UP foods (class in which MSM are present) was higher among men, younger adults, and those with fewer years of formal education^39^. In addition to this study, a systematic review of the global consumption of UP foods observed high variability based on sex and age, with men and young people generally showing higher levels of consumption compared to older individuals^38^.

It is worth mentioning that, in our results, when controlling by age, greater consumption of MSM was also related to greater waist circumference. Waist circumference is strongly associated with all-cause and cardiovascular mortality^40–42^ and is used for cardiovascular risk classification. Consumption of processed meat has also been associated with negative health outcomes, as shown by a meta-analysis of nine prospective cohort studies that estimated that each daily portion of processed meat (the group in which MSM is included) was associated with a 15% increase in the risk of all-cause and cardiovascular mortality^43^.

Consequently, it is necessary to give more attention to the nutritional composition of foods based on MSM. To the best of our knowledge, this is the first study that analyzed nutrients and energy consumption through nutritional analysis. In our results, we have seen a relationship between greater consumption of MSM and greater intake of calories, fats (including total, saturated, monounsaturated and omega-6), carbohydrates and sodium. Furthermore, MSM is not equivalent to manually boned meat. A study evaluating the chemical composition of MSM through density measurements, found that manually boned meat, in relation to all types of MSM, is characterized by higher density, higher protein content and lower sodium, calcium and fat content^2^. Therefore, replacing manually boned meat with MSM cannot be justified as an improvement in nutritional composition. In some parts of the world, such as the European Union, products of animal origin represent 58% of the availability of proteins per capita/day, and meat products constitute the main source of proteins (28 g of protein/person/day), corresponding to 30% of total caloric consumption^44^. These data make us think about the consequences of replacing minimally processed animal proteins with MSM, as was seen in in the present study, where a reduction in the consumption of red and white meat and eggs was seen in individuals who consume more MSM, as the characteristics and protein content contained in MSM are not similar to those in unprocessed or minimally meat or in eggs.

It was also observed in this study that individuals who consume more MSM, in addition to lower consumption of minimally processed animal proteins also have lesser consumption of unprocessed foods, such as fruits, roots and tubers. This demonstrates a tendency towards greater consumption of processed foods in general. We have also found that MSM consumers have greater consumption of corn, which most likely is due to the preparations that accompany some MSM products, namely sausages and frankfurters, such as regional dishes based on corn couscous, widely consumed in the northeast of Brazil. This is an example of the insertion of MSM into traditional culinary preparations and its potential advancement in the population’s eating habits.

Therefore, given all this potential for commercial expansion of MSM, further studies are needed to analyze the impact of its consumption on the population so that it can be promoted and alert to the need to implement new regulations and inspections, which are still scarce due to the lack of knowledge related to this type of food product, both by the scientific community, the society, and the consumers. Little evidence has analyzed its nutritional aspects and effects on health and food consumption, but it is known that this type of product falls within the group of ultra-processed foods, which are widely related to unfavorable health outcomes^45^.

This study has some limitations, such as having been carried out in a single federation state. As the survey was conducted on the population of manufacturing workers, the estimates may not be readily generalizable to other populations. The strengths of our study include it being based on a representative sample of the population obtained with a complex survey design, large sample size, no subject dropouts or refusals to participation, direct interviews by trained nutritionists, and design-based analysis incorporating survey weights.

The results of this study show an association between the consumption of MSM with higher intake of energy, carbohydrates, fats, and sodium, as well as with higher intake of UP products and lower intake of minimally processed/in natura foods. There is tendency for the substitution of protein from unprocessed sources by MSM products and, although they do not have the same nutritional characteristics, their affordable prices combined with a lack of technical information related to quality, have already made them part of the population’s dietary habits.

### Supplementary Information

Supplementary Information.

## Data Availability

Study data are available upon reasonable request to the corresponding author.
